# Efficacy of *Helicobacter pylori* eradication therapy for treatment of functional dyspepsia: A protocol for systematic review and meta-analysis

**DOI:** 10.1097/MD.0000000000026045

**Published:** 2021-05-21

**Authors:** Jue Wang, Sai Gu, Bo Qin

**Affiliations:** Department of Gastroenterology, the First Affiliated Hospital of Chongqing Medical University, Chongqing, China.

**Keywords:** functional dyspepsia, *Helicobacter pylori*, meta-analysis, peptic ulceration

## Abstract

**Objective:**

The effect of *Helicobacter pylori* (*H pylori*) eradication therapy in functional dyspepsia (FD) patients was inconsistent in previously published randomized controlled trials. Therefore, we performed a comprehensive protocol for systematic review and meta-analysis in order to assess whether *H pylori* eradication therapy benefits patients with FD.

**Methods:**

In this systematic review and meta-analysis, we will search Web of Science, Embase, PubMed, Wanfang Data, Medline, Science Direct, Cochrane Library through April, 2021. The protocol was written following the Preferred Reporting Items for Systematic Reviews and Meta-Analyses Protocols. Data extraction was performed independently and only randomized clinical trials were included in this study. The risk of bias assessment was performed using the tool recommended in the Cochrane Handbook for Systematic Reviews of Interventions. All calculations were carried out with Stata 11.0 (The Cochrane Collaboration, Oxford, United Kingdom).

**Results:**

We hypothesized that *H pylori* eradication therapy compared to no eradication therapy has a statistically significant benefit for symptom relief and can also reduce the development of peptic ulcer disease.

**Conclusion:**

This study expects to provide credible and scientific evidence for the efficacy of *H pylori* eradication therapy for patients with FD.

**OSF registration number:**

10.17605/OSF.IO/4EHRB

## 1 Introduction

Functional dyspepsia (FD) is a clinical syndrome defined as the presence of symptoms thought to originate from the gastroduodenal region in the absence of organic, systemic, or metabolic disease likely to explain the symptoms.^[1,2]^ FD is a common morbid condition and has great impact on the quality of life, healthcare usage, and socioeconomic cost. Globally, the prevalence of FD varies between 11% and 29%,^[3]^ and the direct and indirect costs of FD are substantial because of its chronic and recurrent characteristics. The underlying pathophysiology of FD is incompletely understood and may be heterogeneous, possibly including mechanisms such as hypersensitivity to gastric distension, impaired meal accommodation, and delayed gastric emptying.^[4,5]^ According to the Rome III consensus, the main symptoms of FD include bothersome postprandial fullness, early satiety, epigastralgia, and epigastric burning, and FD is further subdivided into two subcategories: meal-induced postprandial distress syndrome (characterized by postprandial fullness and early satiation) and epigastric pain syndrome (characterized by epigastric pain and burning).

The discovery of *Helicobacter pylori* (*H pylori*) triggered the expectation that dyspeptic symptoms could be caused by such a persistent infection in the stomach. This infection was estimated to be about 2.3 fold in patients with FD compared with normal controls and *H pylori* was found in about half of the patients with FD.^[6]^ Therefore, there have been many studies on whether *H pylori* eradication therapy is effective in relieving the symptoms of dyspepsia.^[7,8]^ However, the effect of eradication therapy in FD patients was inconsistent in previously published randomized controlled trials because of variable study designs and follow-up durations. Therefore, we performed a comprehensive protocol for systematic review and meta-analysis in order to assess the overall clinical impact of *H pylori* eradication therapy in patients with FD.

## 2 Materials and methods

### 2.1 Protocol registration

The prospective registration has been approved by the Open Science Framework registries (https://osf.io/4ehrb), and the registration number is 10.17605/OSF.IO/4EHRB. The protocol was written following the Preferred Reporting Items for Systematic Reviews and Meta-Analyses Protocols statement guidelines.^[8]^ Ethical approval is not necessary because this is a meta-analysis.

### 2.2 Study selection

Electronic databases including Web of Science, Embase, PubMed, Wanfang Data, Medline, Science Direct, Cochrane Library were searched in April 2021 by 2 independent reviewers.

The main search strategies were as follows: “*Helicobacter pylori* OR Campylobacter OR *Campylobacter pylori* OR *C pylori* OR Helicobacter infection” AND “treat OR eradication OR eradicating OR therapy OR anti-” AND “dyspepsia OR functional gastrointestinal disorder OR non-ulcer dyspepsia OR functional dyspepsia.” The reference lists of the included studies were also checked for additional studies that were not identified with the database search. There was no restriction in the dates of publication or language in the search.

### 2.3 Inclusion and exclusion criteria

Studies were considered eligible if they met the following criteria:

1. randomized controlled trials;
2. study population of patients with dyspepsia (symptom-based criteria including ROME I, II, III or IV) and *H pylori* infection (^13^C breath test, histology, or rapid urease teat);
3. *H pylori* eradication regimens (dual, triple, quadruple, and sequential therapies) as intervention for treatment group and placebo or other drugs known not to eradicate *H pylori* (no antibiotics or bismuth) as intervention for control groups; and
4. age above 16 years old.

Studies were excluded if they were available only as case reports, comments or letters, biochemical trials, protocols, conference abstracts, reviews, or if predefined outcome data required for analyses were lacking.

### 2.4 Data extraction

Two investigators reviewed all the titles and abstracts independently. Data were extracted from eligible full-text studies. The data included study population, demographical characteristics, year of publication, country, age, gender, *H pylori* eradication regimens, duration of follow-up, and study outcomes. The primary outcome for this study was the rate of successful treatment (presence of no more than mild pain or discomfort after treatment). The secondary outcomes were improvement of dyspepsia at short-term (<1 year) and long-term (≥1 year) follow-up, improvement in quality of life, incidence of peptic ulceration during follow-up, development of treatment-related adverse events, and histologic resolution of chronic gastritis. Data extraction was performed independently, disagreements between the 2 reviewers were discussed and, if necessary, the third author was referred to for arbitration. If the data were missing or could not be extracted directly, authors were contacted by email.

### 2.5 Quality evaluation

The risk of bias assessment of the included articles was performed by 2 authors independently using the tool recommended in the Cochrane Handbook for Systematic Reviews of Interventions^[9]^ which contains random sequence generation, allocation concealment, blindness, incomplete outcome data, selective outcome reporting, and other biases. Additionally, each of the aspects was ranked low risk of bias, high risk of bias, and unclear risk of bias. The evidence grade was assessed using the guidelines of the GRADE (Grading of Recommendations, Assessment, Development, and Evaluation) working group including the following items: risk of bias, inconsistency, indirectness, imprecision, and publication bias. The recommendation level of evidence was classified into the following categories:

1. high, which means that further research is unlikely to change confidence in the effect estimate;
2. moderate, which means that further research is likely to significantly change confidence in the effect estimate but may change the estimate;
3. low, which means that further research is likely to significantly change confidence in the effect estimate and to change the estimate; and
4. very low, which means that any effect estimate is uncertain. GRADE pro Version 3.6 software is used for the evidence synthesis.

### 2.6 Statistical analysis

The risk differences with 95% confidence intervals were calculated for dichotomous data, and the weighted mean difference with 95% confidence intervals was calculated for the continuous data. Heterogeneity between the studies was assessed by the χ2 test (significant level of *P* < .10) and the *I*^2^ statistic (*I*^2^ > 50% indicating significant heterogeneity). The results were pooled using the fixed-effect model for *P* > .10 and *I*^2^ < 50% or the random-effect model for *P* < .10 and *I*^2^ > 50%. If significant heterogeneity is found, we will try to explore the source of heterogeneity by subgroup analysis. Publication bias was assessed by drawing contour-enhanced funnel plots. When these plots were not obviously asymmetric, we considered that publication bias was absent. All calculations were carried out with Stata 11.0 (The Cochrane Collaboration, Oxford, United Kingdom).

## 3 Results

We hypothesized that *H pylori* eradication therapy compared to no eradication therapy has a statistically significant benefit for symptom relief and can also reduce the development of peptic ulcer disease.

## 4 Discussion

*H pylori* is strongly associated with many diseases, including FD, gastric or duodenal ulcer, gastric cancer, and gastric mucosa-associated lymphoid tissue lymphoma.^[10–12]^ Most patients with *H pylori* infection have asymptomatic gastritis, and experience variable clinical symptoms depending on bacteria, host, and environmental factors. Whether *H pylori* infection delays gastric emptying is unclear,^[13,14]^ but *H pylori* appears to alter gastric acid production by changing gastrin and somatostatin secretion.^[15]^ Abnormal gastric acid secretion causes mainly dysmotility-like, dyspeptic symptoms.^[16]^ Duodenal acid exposure indirectly induces fullness, bloating, and epigastric pain by suppressing antral contractions, which may contribute to delayed gastric emptying.^[17]^ Previous studies on the effect of *H pylori* eradication on FD are conflicting. This study expects to provide credible and scientific evidence for the efficacy of *H pylori* eradication therapy for patients with FD.

## Author contributions

**Conceptualization:** Sai Gu.

**Data curation:** Sai Gu.

**Funding acquisition:** Bo Qin.

**Writing – original draft:** Jue Wang.

**Writing – review & editing:** Bo Qin.
